# Hydrodynamic Choreographies of Microswimmers

**DOI:** 10.1038/s41598-018-21832-w

**Published:** 2018-02-27

**Authors:** Mehdi Mirzakhanloo, Mir Abbas Jalali, Mohammad-Reza Alam

**Affiliations:** Department of Mechanical Engineering, University of California, Berkeley, CA 94720 USA

## Abstract

We unveil orbital topologies of two nearby swimming microorganisms using an artificial microswimmer, called *Quadroar*. Depending on the initial conditions of the microswimmers, we find diverse families of attractors including dynamical equilibria, bound orbits, braids, and pursuit–evasion games. We also observe a hydrodynamic slingshot effect: a system of two hydrodynamically interacting swimmers moving along braids can advance in space faster than non-interacting swimmers that have the same actuation parameters and initial conditions as the interacting ones. Our findings suggest the existence of complex collective behaviors of microswimmers, from equilibrium to rapidly streaming states.

## Introduction

Microorganisms including all species of bacteria, protozoa, and also some of alga, are playing an important role in recycling nutrients in the Earth’s ecosystems^1,2^. A recent report by National Science Foundation (NSF) estimates the presence of about one trillion species right now on the Earth, that only one-thousandth of one percent of which have been studied^3^. This fact has put the investigation of natural or artificial microswimmers under the spotlight^4–7^.

Swimming microorganisms in nature nearly always come in groups, and understanding their collective behaviors in the presence of hydrodynamic interactions requires multi-scale models^8–10^. The main challenge in developing statistical and continuum models is how we deal with collisional and relaxation processes, which are basically determined by two-body interactions^11^. This is exacerbated by the long range nature of hydrodynamic interactions at low Reynolds number conditions that makes the investigation of the swarm dynamics of microswimmers substantially different from other well-studied swarms. Specifically, swimmers at small scales strongly affect (at distances several body-length away) their fluidic environment and hence their nearby swimmers (compare this with, say, Quadcopters whose influence on nearby copters is limited to a fraction of their body length). An interesting example, showing the significance of hydrodynamic interactions, is that they may trigger the locomotion of otherwise non-swimming reciprocal swimmers^12,13^.

Prior observations have revealed a glimpse of these complex behaviors in nature^14–18^. For instance, the parallel motion of two flagella-driven bacteria have been shown to be unstable^14^, two nematodes tied to a wall from one end eventually get entangled^15^, and two Paramecia avoid each other solely due to hydrodynamic interactions^16^. Previous studies have addressed two-body dynamics using simple minimal model swimmers^19–26^ and have been able to report few basic behaviors. For example, two puller-type squirmers experience a significant change in their orientations after an encounter^19^, which together with the swimmers’ inertial effects, causes hydrodynamic attraction^20^. For spherical swimmers with spatially confined circular trajectories, the only possible long-time cell–cell interaction is either an attraction or repulsion^21^, whereas two rigid helices do not attract or repel each other while rotating in-phase^22^. Another example is a systems of two linked-sphere swimmers^27^ that may converge, diverge oscillate or stay parallel to each other^23,24^.

Recent experiments, nevertheless, uncover more complex flow fields around flagellated microorganisms than what had been previously thought^28^. Specifically, flagellated microorganisms such as Chlamydomonas reinhardtii induce an oscillatory flow field that alternates between the flow fields of basic puller- and pusher-type swimmers^28,29^, complicating the nature of two- or multi-body interactions of such microorganisms. To gain insight into the two- and three-dimensional interactions of microorganisms, we simulate them using the Quadroar swimmer whose flow field^30^ is similar to that of *C. reinhardtii*^28,29,31^. One of the major advantages of the Quadroar as an artificial microswimmer is that it consists of rotary disks and only one reciprocating actuator. This remarkably simplifies the realization of the Quadroar as linear actuators (in all scales) are hard to fabricate and assemble. In nano-scales, the science and engineering of making molecular rotary units have leapt forward and claimed the Nobel prize in Chemistry in 2016^32^, and molecular-scale linear actuators can, in principle, be made of certain proteins^33^. The Quadroar is highly controllable and has full three-dimensional maneuverability. It can therefore track any prescribed spatial path^33^. This has been a challenge in the design of medical microbots^34^ that makes a submillimeter-scale Quadroar also a suitable candidate for various biological applications^35^ such as drug delivery or autonomous surgery^34^. In macro scales, the Quadroar can be hired as a robotic swimmer^36,37^ for inspection missions in highly viscous fluid reservoirs^38^.

In this study, we use the Quadroar swimmer and show that two model micro-swimmers in the Stokes regime–that generate flow fields resembling that of C. reinhardtii–have a rich two-body dynamics. Unlike other existing theoretical models that try to simulate the swimming mechanism of specific microorganisms^39^, the Quadroar is designed to induce an oscillatory flow field with anterior, side and posterior vortices in its surrounding^30^. Therefore, complex interactions that we find in the phase-space of two swimmers are generic characteristics of microorganisms generating anterior, side and posterior vortices.

## Kinematics and Numerical Framework

**Figure 1:**
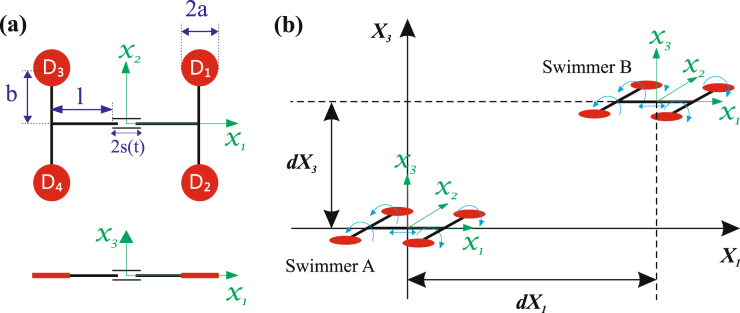
Quadroar, a low-Reynolds-number swimmer whose flow field resembles that of *C. reinhardtii*. (**a**) Geometry of the Quadroar swimmer. (**b**) Relative initial positions of two planar swimmers considered in this study.

The Quadroar consists of an I-shaped frame including an active chassis and two axles of length 2*b* (see Fig. 1). Each axle at its two ends is connected to two disks of radii *a*. The length of the chassis is variable and is equal to 2*l* + 2*s*(*t*) where *s*(*t*) is the contribution from the expansion/contraction of a linear actuator installed in the middle of the chassis. The angular position of each disk *D*_*n*_ (*n* = 1, …, 4) with respect to the leg of its axle is denoted by − *π* ≤ *ϑ*_*n*_ ≤ *π*. We define a body-fixed Cartesian coordinate system (*x*_1_, *x*_2_, *x*_3_) with its origin at the geometrical center of the frame. The (*x*_1_, *x*_2_)-plane lies in the plane of the swimmer and the *x*_1_-axis is along the chassis. We also define a global Cartesian coordinate system (*X*_1_, *X*_2_, *X*_3_) as is shown in Fig. 1(b). The body-fixed coordinates *x*_*i*_ are related to the global coordinates *X*_*i*_ (*i* = 1, 2, 3) through a transformation matrix *R* that depends on three orientation (Euler’s) angles of the swimmer. We assume that the influence of each disk on its surrounding environment can be modeled as a point force and a point torque^40^.

For each of the two swimmers, *j* = *A*, *B*, and for each of their four disks *n* = 1, …, 4, the point forces (*f*_*jn*_) and torques (***τ***_*jn*_), expressed in the global coordinate frame are given by^30^

1 $${{\boldsymbol{f}}}_{jn}=\mu {{\bf{R}}}_{j}^{T}\cdot {{\bf{K}}}_{jn}\cdot {{\bf{R}}}_{j}\cdot ({v}_{j,c}+{v}_{jn}-{{\boldsymbol{u}}}_{jn}),$$

2 $${{\boldsymbol{\tau }}}_{jn}=\mu {\bf{G}}\cdot ({{\boldsymbol{\omega }}}_{j,{\rm{b}}{\rm{o}}{\rm{d}}{\rm{y}}}+{{\boldsymbol{\omega }}}_{jn}-{{\boldsymbol{\Omega }}}_{jn}),$$

where *μ* denotes dynamic viscosity of the surrounding fluid; *v*_*jn*_ and ***ω***_*jn*_ are the linear and angular velocities of each disk with respect to the swimmer’s hydrodynamic center and body-fixed coordinate frame, respectively; *v*_*j*,*c*_ is the absolute velocity of the hydrodynamic center and ***ω***_*j*,b*ody*_ is the angular velocity of the *j*th swimmer expressed in terms of Euler’s angles; ***u***_*jn*_ and 2**Ω**_*jn*_ are the velocity and vorticity fields of the fluid at the center of each disk; ***G*** is the isotropic rotation tensor and **K**_*jn*_ is the translation tensor corresponding to disk *n* of swimmer *j*, defined as^33^:

3 $${\bf{G}}=\frac{32}{3}{a}^{3}{\boldsymbol{I}},\quad \quad {{\bf{K}}}_{jn}=\frac{8}{3}a[\begin{array}{ccc}5-\,\cos (2{\vartheta }_{jn}) & 0 & \sin (2{\vartheta }_{jn})\\ 0 & 4 & 0\\ \sin (2{\vartheta }_{jn}) & 0 & 5+\,\cos ({\vartheta }_{jn})\end{array}],$$

Since a self-propelled swimmer in the Stokes regime is force-free and torque-free, we must have ${\sum }_{n=1\,}^{4}{f}_{jn}\,=\,0$ and also ${\sum }_{n=1}^{4}[({{\bf{R}}}_{j}^{T}\cdot {{\boldsymbol{r}}}_{jn})\times {f}_{jn}+{{\boldsymbol{\tau }}}_{jn}]=0$ for *j* = *A*, *B*. These four sets of vectorial equations (two vectorial equations for each swimmer) require the values of velocities ***u***_*jn*_ and spins **Ω**_*jn*_ to be complete and solvable for *v*_*c*_ and ***ω***_body_. The linear nature of the Stokes equation allows us to invoke superposition and obtain^30,40^:

4 $$\begin{array}{c}{c}_{0}\,{{\boldsymbol{u}}}_{jn}=\sum _{k=1,k\ne n}^{4}(\frac{{f}_{jk}}{{z}_{kn,j}}+\frac{{f}_{jk}\cdot {{\bf{X}}}_{kn,j}}{{z}_{kn,j}^{3}}{{\bf{X}}}_{kn,j}+\frac{{{\boldsymbol{\tau }}}_{jk}\times {{\bf{X}}}_{kn,j}}{{z}_{kn,j}^{3}})+\sum _{k=1}^{4}(\frac{{f}_{ik}}{{z}_{kn,ij}}+\frac{{f}_{ik}\cdot {{\bf{X}}}_{kn,ij}}{{z}_{kn,ij}^{3}}{{\bf{X}}}_{kn,ij}+\frac{{{\boldsymbol{\tau }}}_{ik}\times {{\bf{X}}}_{kn,ij}}{{z}_{kn,ij}^{3}}),\\ 2{{\boldsymbol{\Omega }}}_{jn}={\boldsymbol{\nabla }}\times {{\boldsymbol{u}}}_{jn},\end{array}$$

where *c*_0_ = 8*πμ*, ${{\boldsymbol{X}}}_{kn,j}={{\bf{R}}}_{j}^{T}\cdot ({{\boldsymbol{r}}}_{jn}-{{\boldsymbol{r}}}_{jk})$ and ${{\boldsymbol{X}}}_{kn,ij}=({{\boldsymbol{X}}}_{c,j}+{{\bf{R}}}_{j}^{T}\cdot {{\boldsymbol{r}}}_{jn})-({X}_{c,i}+{{\bf{R}}}_{i}^{T}\cdot {{\boldsymbol{r}}}_{ik})$. The scalars *z*_*kn*,*j*_ and *z*_*kn*,*ij*_ are the magnitudes of the vectors *X*_*kn*,*j*_ and *X*_*kn*,*ij*_, respectively, and *r*_*jn*_ denotes the position vector of the *n*th disk in the swimmer’s local coordinate frame. In all expressions we have *i*,*j* = *A*, *B* with the condition *i* ≠ *j* in each expression.

We assume that disks *D*_*n*_ (*n* = 1, …, 4), of each swimmer *j* = *A*, *B* are spinning with angular velocities ${\dot{\vartheta }}_{j1}={\dot{\vartheta }}_{j2}={c}_{0}{\omega }_{s}$ and ${\dot{\vartheta }}_{j3}={\dot{\vartheta }}_{j4}=-{c}_{0}{\omega }_{s}+\delta \omega$ where *δω* is a detuning parameter, and the length of the linear actuator at the middle of the chassis varies according to *s*(*t*) = *s*_0_[1 − cos(*ω*_*s*_*t*)]/2. Throughout our simulations we set *a* = 1, *b*/*a* = 4, *l*/*a* = 4, *s*_0_/*l* = 1/2, and *ω*_*s*_ = 1. The characteristic time scale of the two-body system is *T*_*s*_ = 2*π*/*ω*_*s*_. The parameter *c*_0_ affects both the swimmer’s dynamics and flow field around it. For *c*_0_ ≈ 0.5, it has been shown^30^ that the flow field induced by the Quadroar closely resembles that of C. reinhardtii alga^29^. Our numerical experiments show that the resemblance holds for almost any *c*_0_ ≥ 1. This is similar to the recent experimental observations^41^ that swimming speed or beat frequency do not have a considerable effect on bioconvection behavior of C. reinhardtii cells. The similarity in behaviors for this broad range of *c*_0_, which even includes the single-frequency case (i.e., *c*_0_ = 1), adequately addresses the concern about whether any of emerging dynamical regimes is affected by the presence of two different frequencies. This further highlights the significance of having an oscillatory flow field. To speed up numerical simulations, we set *c*_0_ = 50. For individual swimmers, nonzero values of *δω* significantly increase the number of orbital families, and in some cases lead to densely interwoven quasi-periodic rosette-shaped trajectories capable of inducing chaotic mixing in the surrounding environment^30^. Here, to focus on the basics of mutual interactions, we consider *δω* = 0. The results of this study are still valid for small *δω*, but start to deviate and become more involved as *δω* increases. Note that *a* = 1 *μm* leads to a Quadroar of ∼8–12 *μm*, which is similar to the size of a C. reinhardtii cell. Moreover, setting *c*_0_ = 50 results in the frequency of 50 *Hz* for the disks, reminiscent of the flagella beat frequency for a C. reinhardtii cell^29^. For a more detailed description of the flow field around an isolated Quadroar the reader is referred to earlier publications on the Quadroar dynamics^30^.

## Results

**Figure 2:**
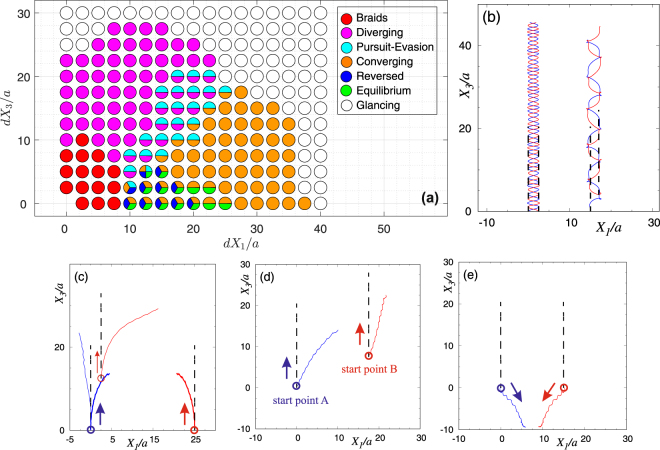
Coupled dynamics of two initially co-moving microswimmers as a function of their initial relative positions. (**a**) The orbital/trajectory structure in the parameter space (*dX*_1_, *dX*_3_). Trajectories belong to two general families: braids (**b**) and non-orbiting paths such as diverging (thin lines) and converging (thick lines) (**c**), pursuit-evasion (**d**), and reversed motion that ends up at a dynamical equilibrium state (**e**). Orbits corresponding to the initial conditions marked as “glancing” in panel (a) exhibit small drifts from straight paths. All simulations have been performed over the time span of [0, 10 *T*_*s*_]. The trajectories of the swimmers have been plotted in blue and red colors. The launch direction of each swimmer and also the starting positions of swimmers *A* and *B* are marked in panels (c)–(e). The swimmers would move on dashed lines in the absence of hydrodynamic interactions, i.e. when swimming alone. Since simulation times are identical, the longer the travelled distances, the faster the swimmers’ motions.

**Figure 3:**
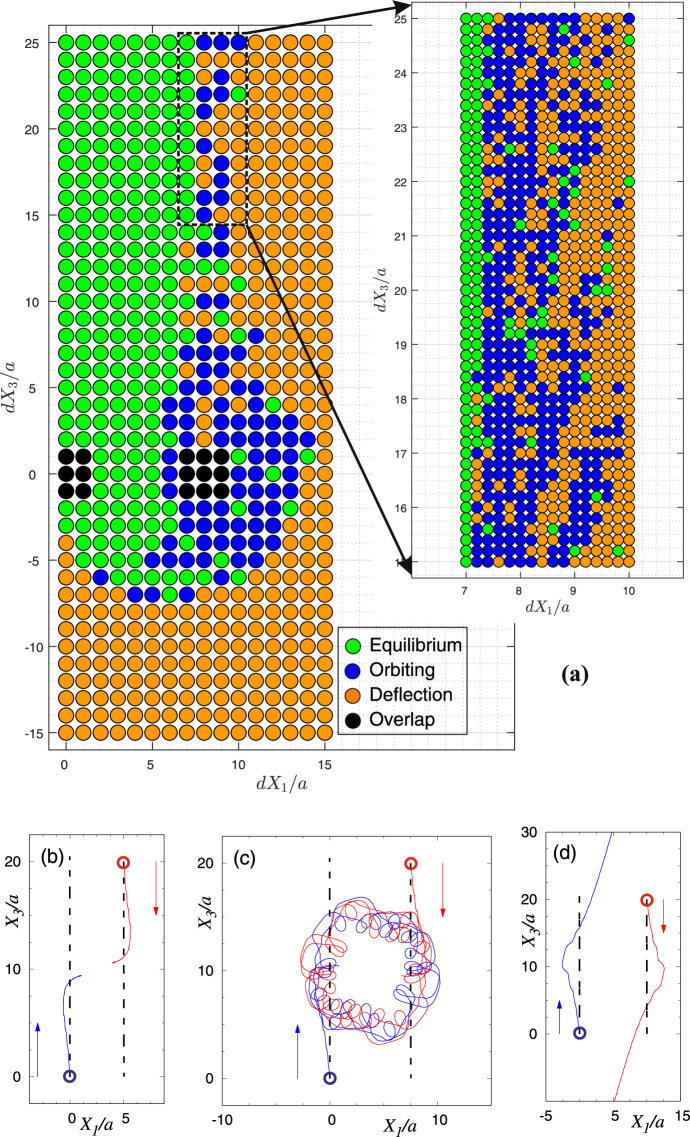
Coupled dynamics of two swimmers initially moving in opposite directions. (**a**) The parameter space of possible trajectories. We have used finer grids in the zoomed area. (**b**) Moving towards an equilibrium state. (**c**) Capture into a bound quasi-periodic orbit. (**d**) Deflection of trajectories after a close encounter. In panels (b)–(d), the trajectories of the swimmers have been shown by blue and red solid lines. The arrows denote the launch directions of the swimmers, and the circles mark their starting positions. The swimmers would follow dashed lines in the absence of hydrodynamic interactions.

Two of our swimmers, depending on their relative initial locations (*dX*_1_, *dX*_3_) portray a range of various trajectories as a result of their mutual hydrodynamic interactions. These trajectories range from converging, diverging, and oscillatory motions (which are also seen in other artificial microswimmers^23^), to forming *braids* [Fig. 2(b)], and even *dynamical equilibria* [Figs 2(e)and 3(b)] which, to the best of our knowledge, have never been observed in low-Reynolds-number swimming. We also report capture into *bound orbits* [Fig. 3(c)] for two interacting microorganisms swimming in an infinite unbounded fluid. Interestingly, a similar behavior is observed in the lab for two Volvox colonies attracted by the chamber ceiling^42^.

In order to systematically study different possibilities of two-swimmer wiggling, induced by hydrodynamic interactions, we simultaneously consider the effects of swimming direction and relative initial locations, which also cover phase shift effects. Since there is no explicit time dependency in the Stokes equation, swimmers with an arbitrary phase shift between them (as a result of being launched at different times) can be considered as two swimmers with initial locations described at the moment that the second swimmer is turned on. For simplicity, we focus on the planar phase space. Nevertheless, our findings can be inherently generalized to 3D space. Our study has also been conventionally arranged in two general categories: (i) the two swimmers are released in the same direction such that their initial *x*_3_-axes are parallel and both aligned with the positive *X*_3_-axis (cf. Fig. 1), and (ii) the two swimmers are initially facing opposite directions such that at *t* = 0 the following conditions hold: ${\hat{{\boldsymbol{x}}}}_{3A}$ ⋅ ${\hat{{\boldsymbol{X}}}}_{3}=1$, and ${\hat{{\boldsymbol{x}}}}_{3B}$ ⋅ ${\hat{{\boldsymbol{X}}}}_{3}=-1$, where the hat sign denotes unit vector. The resulting parameter space for each of these general cases is still valid for small perturbations. For larger perturbations, however, the parameter space starts to deviate from the presented plot and gradually tends to that of the other general case. For example, by changing the relative angle between the swimmers’ initial *x*_3_-axes from zero to *π*, the corresponding parameter space diagram will gradually transform from Fig. 2a (swimming in the same direction) to Fig. 3a (swimming in opposite direction).

**Figure 4:**
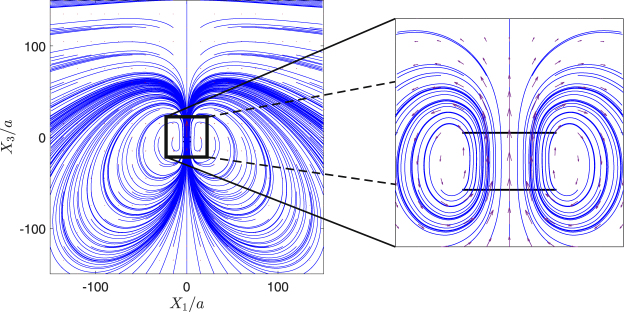
Streamlines of the flow field induced by two microswimmers having initial velocities of the same magnitude and swimming in the same direction. The snapshot has been taken at *t*/*T*_*s*_ = 0.05, and relative initial positions are *dX*_1_/*a* = 0 and *dX*_3_/*a* = 5, which correspond to the braid-like trajectories. Simulation parameters are the same as the ones used in Fig. 2. Rectangular selection (magnified in the right panel) shows the area near the two swimmers, and black bars in the right panel are the swimmers’ chassis.

The parameter space for the trajectories of two swimmers released parallel and in the same direction [case (i)] is displayed in Fig. 2(a) with sample trajectories demonstrated in Fig. 2(b–e). In these figures, the swimmers would follow dashed lines in the absence of hydrodynamic interactions. If the two swimmers are released close to each other, and depending on their relative locations, they form a variety of braids with different shapes [Fig. 2(b)]. Interestingly, we find that forward translational motion along a braid is faster, sometimes by a factor of two, than the motion of individual swimmers in the absence of hydrodynamic interactions. This phenomenon, which we refer to as *hydrodynamic slingshot effect*, can be easily deduced from Fig. 2(b): two hydrodynamically interacting swimmers advance along braids, and therefore in space (motion along colored lines), faster than non-interacting swimmers (moving along dashed lines) whose actuation parameters and initial conditions exactly match those of the interacting ones. The slingshot effect is caused by a synergistic process: each swimmer induces an advection field that sums with the relative velocity of its companion swimmer with respect to the background fluid, boosting the absolute velocity of the companion swimmer. Figure 4 demonstrates a snapshot of the flow field and corresponding streamlines induced by the system of two swimmers advancing along a braid-like trajectory. It shows how each swimmer induces an advection field at the geometric center of the other swimmer, boosting its absolute velocity. The net flow field could also be described as a constructive interference of the two swimmers’ flow fields (see Fig. 4). The resulting net flow field is similar to the one induced by a single Quadroar swimmer^33^ but with a more powerful propellers (higher values of *c*_0_).

**Figure 5:**
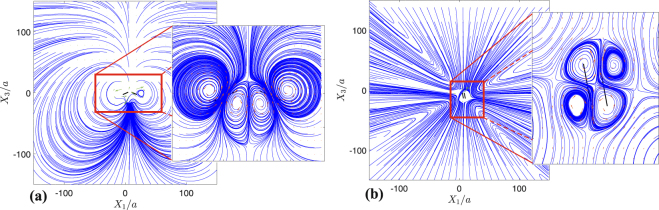
Streamlines of the flow field induced by two microswimmers having initial velocities of the same magnitude and swimming in same directions. The swimmers’ relative initial positions are *dX*_1_/*a* = 15 and *dX*_3_/*a* = 0. Snapshots have been taken at *t*/*T*_*s*_ = 1 (**a**), and *t*/*T*_*s*_ = 40 (**b**). At full equilibrium state, corresponding to *t*/*T*_*s*_ = 40, four vortices have been formed by the swimmers. Simulation parameters are same as the ones used in Figs 2 and 3. Thick solid black bars represent the side views of the chassis, and the area near the two swimmers have been magnified in right panels.

Other families of trajectories that we observe for interacting swimmers belong to a general family of non-orbiting paths including diverging and converging trajectories [Fig. 2(c)]. Non-orbiting paths may occur as pursuit-evasion games when one of the swimmers chases the other one [Fig. 2(d)]. The most interesting non-orbiting path that we have found happens when the swimmers get in a reverse motion [Fig. 2(e), colored dark blue in Fig. 2(a)], eventually reaching to a *dynamical equilibrium*. In dynamical equilibrium states, the swimmers’ propellers are working continuously and their flow fields form a saddle structure (Fig. 5). The net flow of the saddle structure is zero, so follows the equilibrium state. In the space between the swimmers, fluid is pumped out in a direction almost parallel to the chassis of both swimmers, and is sucked back normal to the chassis. Four prominent vortices are formed around the propellers of the swimmers. These vortices are enclosed by a large-scale hyperbolic structure. Our long-term simulations show that dynamical equilibria are stable to small perturbations. This is a counter-intuitive property because the existence of hyperbolic structures usually implies local instability. The existence of dynamical equilibria for *N* > 2 swimmers is an unsolved problem, whose solution can sharpen our understanding of bacterial clustering and motile cell accumulations^43,44^.

If the initial distance of the swimmers is large enough, their hydrodynamic interaction will be very small, drifting the swimmers slightly off their straight trajectories. We have also observed a switch between different trajectories as the two-body system evolves: a converging motion may end up in an equilibrium state, or pursuit-evasion game may bifurcate to either of converging or diverging paths. In the parameter space, we have marked these cases with two or more colors [Fig. 2(a)].

Two swimmers starting their motions in opposite directions [case (ii)] exhibit different orbital topologies from what we observed for co-directional ones. Their two-body dynamics depends on the impact parameter *dX*_1_ [Fig. 3(a)]. When the impact parameter is relatively small ($d{X}_{1}/a\lesssim 5$) and the swimmers initially move towards each other, *dX*_3_ > 0, we always obtain an equilibrium state [Fig. 3(b)]. Similar to the equilibria of case (i), the actuators of the swimmers are operational at the equilibrium state and energy is consumed only for flow generation (and not translation). For large impact factors, $d{X}_{1}/a\gtrsim 15$, trajectories are deflected similar to the lensing/refraction of light rays [Fig. 3(d)]. The deflecting trajectories have also been observed in linked-sphere swimmers^24,27^. Our results are in agreement with the angle-preserving confrontation of two T-dual swimmers^25^. For intermediate impact factors, we observe a *capture* phenomenon as the microswimmers begin to orbit each other after a translational phase [Fig. 3(c)]. It is noted that capture into a quasi-periodic orbit is a transitional state between dynamical equilibria and deflecting trajectories. Such transitional states fill a complex fractal-shaped region of the parameter space, showing high degree of sensitivity to initial conditions [see the zoomed-in box in Fig. 3(a)] with the dominant length-scale of a disk radius. This result suggests the existence of highly chaotic *N*-body systems of swimming microorganisms. Although details of trajectories in an orbiting motion can be complex, the bounded nature of the overall two-body motion in an infinite fluid domain is a unique physical process, for which many applications can be sought. Examples include mixing by microswimmers and trapping microorganisms by artificial microswimmers.

## Conclusions and Discussion

Here we have shown that two microswimmers in Stokes regime can stop each other by forming a dynamical equilibrium in an infinite fluid domain. Furthermore, depending on where the two swimmers are released, they may also get trapped into bounded orbits and revolve about each other indefinitely. We have systematically studied the entire phase space of a hydrodynamically interacting two-swimmer system, and identified the basins of dynamical equilibria and periodic orbits in the parameter space. We also found other diverse sets of orbits including closely winding braids, and pursuit–evasion dynamics. Sensitivity to initial conditions, slingshot effect for motions along braids, dynamical equilibria, and capture into bound orbits, as demonstrated in this study, can have unexpected implications to motion of microorganisms. Nonlocal models of passive and active stresses due to hydrodynamic and steric interactions^9^ will then need modifications as diffusion in the phase space cannot be modeled only as a function of macroscopic streaming velocity.

## Methods

### Numerical Techniques

At each time step, we first substitute 1 and 2 into the force and torque balance equations. Then, together with equations presented in 4, the system is solved for the 20 vectorial (60 scalar) unknowns: *v*_*j*,*c*_, ***ω***_*j*,body_, ***u***_*jn*_ and **Ω**_*jn*_ (*j* = *A*, *B*; *n* = 1, …, 4). We then find the position and orientation of each swimmer by integrating *v*_*j*,*c*_ and ***ω***_*j*,body_ in time. The angular velocity of each swimmer, denoted by ***ω***_*j*,body_, is related to the yaw-pitch-roll sequence of Euler’s angles ***α*** = (*ϕ*, *θ*, *ψ*) through

5 $${\dot{{\boldsymbol{\alpha }}}}_{j}={{\boldsymbol{T}}}_{j}^{-1}\cdot {{\boldsymbol{\omega }}}_{j,{\rm{b}}{\rm{o}}{\rm{d}}{\rm{y}}},\quad T=[\begin{array}{ccc}1 & 0 & -sin(\theta )\\ 0 & cos(\varphi ) & cos(\theta )sin(\varphi )\\ 0 & -sin(\varphi ) & cos(\theta )cos(\varphi )\end{array}].$$

Since there is a coordinate-type singularity in *T*, when *θ* = ±*π*/2, all computations have been carried out in the space of unit quaternions ***q***, and then outputs are mapped back onto the space of Euler’s angles ***α***^33^:

6 $${\dot{{\boldsymbol{q}}}}_{j}=\frac{1}{2}[\begin{array}{cccc}0 & -{\omega }_{1} & -{\omega }_{2} & -{\omega }_{3}\\ {\omega }_{1} & 0 & {\omega }_{3} & -{\omega }_{2}\\ {\omega }_{2} & -{\omega }_{3} & 0 & {\omega }_{1}\\ {\omega }_{3} & {\omega }_{2} & -{\omega }_{1} & 0\end{array}]{{\boldsymbol{q}}}_{j},\quad {{\boldsymbol{\omega }}}_{j,{\rm{b}}{\rm{o}}{\rm{d}}{\rm{y}}}=({\omega }_{1},{\omega }_{2},{\omega }_{3}).$$

### Three-dimensional Beads Model Simulation

**Figure 6:**
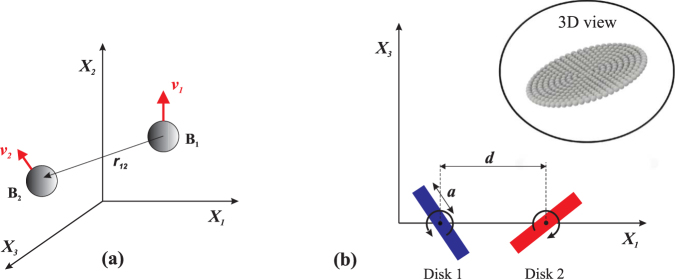
(**a**) Schematic representation for the basic idea of beads model. Beads *B*_1_ and *B*_2_ are moving with absolute velocities *v*_1_ and *v*_2_ with respect to the stationary reference frame fixed to the fluid at infinity. (**b**) Schematics of the 3D beads model realization of two nearby interacting disks of radius *a* and distance *d* apart. The inset shows the three-dimensional view of a single disk composed of 331 spherical beads.

In order to validate our models of the point forces and torques of the disks, we develop a full three-dimensional beads realization of the disks^45,46^. We first briefly explain the concept of beads model using Fig. 6a, then compare the results of our numerical method with those of beads model simulation.

A single spherical bead, moving with velocity *v*_0_, in Stokes regime induces a well-known velocity field in the surrounding fluid. For an arbitrary point in cylindrical coordinate system, this velocity field in radial and tangential directions is given by:

7 $${v}_{r}=(\frac{3{R}_{0}}{2r}-\frac{{R}_{0}^{3}}{2{r}^{3}}){v}_{0}\,cos\theta ,\quad {v}_{\theta }=-(\frac{3{R}_{0}}{4r}+\frac{{R}_{0}^{3}}{4{r}^{3}}){v}_{0}\,sin\theta ,$$

where *R*_0_ is the radius of the bead, and *r****e***_*r*_ is the position vector of the arbitrary point with respect to the bead’s center. Figure 6a demonstrates two beads *B*_1_ and *B*_2_, which are moving with absolute velocities *v*_1_ and *v*_2_ in the stationary frame. With respect to the background fluid, *B*_1_ (*B*_2_) has a hydrodynamic velocity *v*_1*H*_ (*v*_2*H*_), and thus induces a velocity field *v*_1*H*,2_ (*v*_2*H*,1_) at the position of *B*_2_ (*B*_1_). So, the hydrodynamic velocity of each bead is given by the following implicit formula:

8 $${v}_{1H}={v}_{1}-{v}_{2H,1},\quad {v}_{2H}={v}_{2}-{v}_{1H,2}.$$

Generalization of this simple idea, in order to formulate hydrodynamics of a system composed of *N* beads, results in the following system of linear algebraic equations:

9 $${v}_{iH}+\sum _{j=1,j\ne i}^{N}{v}_{jH,i}={v}_{i},\quad i\in \{1,\cdots ,N\}.$$

The velocity *v*_*jH*,*i*_, which is induced at the position of *B*_*i*_ due to the motion of *B*_*j*_, follows from equation (6) as:

10 $${v}_{r-jH,i}=(\frac{3{R}_{0}}{2{r}_{ji}}-\frac{{R}_{0}^{3}}{2{r}_{ji}^{3}}){v}_{jH}\,cos{\theta }_{ji},\quad {v}_{\theta -jH,i}=-(\frac{3{R}_{0}}{4{r}_{ji}}+\frac{{R}_{0}^{3}}{4{r}_{ji}^{3}}){v}_{jH}\,sin{\theta }_{ji},$$

where *r*_*ji*_ = |***r***_*i*_ − ***r***_*j*_|, ***r***_*i*_ and ***r***_*j*_ are position vectors of beads *B*_1_ and *B*_2_, and *θ*_*ji*_ is defined by $cos{\theta }_{ji}=\frac{{{\boldsymbol{r}}}_{ji}\cdot {{\boldsymbol{v}}}_{jH}}{{{\boldsymbol{r}}}_{ji}{{\boldsymbol{v}}}_{jH}}$. To put the system of equations (8) into the standard format of ${\mathscr{A}}{\mathscr{X}}={\mathscr{B}}$, the general hydrodynamic relations between beads is defined here as:

11 $${v}_{m,n}={{\boldsymbol{A}}}_{mn}{v}_{m},\quad {{\boldsymbol{A}}}_{mn}=[{S}_{3\times 1}^{(1)},{S}_{3\times 1}^{(2)},{S}_{3\times 1}^{(3)}],\quad \{m,\,n\}\in \{1,\cdots ,N\}.$$

${S}_{3\times 1}^{(k)}$ for each *k* ∈ {1, 2, 3} is a column vector given by:

12 $${S}_{3\times 1}^{(1)}=(\frac{3{R}_{0}}{2{r}_{mn}}-\frac{{R}_{0}^{3}}{2{r}_{mn}^{3}})[\begin{array}{c}{x}^{2}\\ xy\\ xz\end{array}]-(\frac{3{R}_{0}}{4{r}_{mn}}+\frac{{R}_{0}^{3}}{4{r}_{mn}^{3}})\sqrt{\frac{{r}_{mn}^{2}-{x}^{2}}{{y}^{2}+{z}^{2}}}[\begin{array}{c}-({y}^{2}+{z}^{2})\\ xy\\ xz\end{array}],$$

13 $${S}_{3\times 1}^{(2)}=(\frac{3{R}_{0}}{2{r}_{mn}}-\frac{{R}_{0}^{3}}{2{r}_{mn}^{3}})[\begin{array}{c}yx\\ {y}^{2}\\ yz\end{array}]-(\frac{3{R}_{0}}{4{r}_{mn}}+\frac{{R}_{0}^{3}}{4{r}_{mn}^{3}})\sqrt{\frac{{r}_{mn}^{2}-{y}^{2}}{{x}^{2}+{z}^{2}}}[\begin{array}{c}yx\\ -({x}^{2}+{z}^{2})\\ yz\end{array}],$$

14 $${S}_{3\times 1}^{(3)}=(\frac{3{R}_{0}}{2{r}_{mn}}-\frac{{R}_{0}^{3}}{2{r}_{mn}^{3}})[\begin{array}{c}zx\\ zy\\ {z}^{2}\end{array}]-(\frac{3{R}_{0}}{4{r}_{mn}}+\frac{{R}_{0}^{3}}{4{r}_{mn}^{3}})\sqrt{\frac{{r}_{mn}^{2}-{z}^{2}}{{x}^{2}+{y}^{2}}}[\begin{array}{c}zx\\ zy\\ -({x}^{2}+{y}^{2})\end{array}],$$

where ***r***_*mn*_ = ***r***_*n*_ − ***r***_*m*_ = (*x*, *y*, *z*). Applying this representation to the general formulation of the system of *N* beads presented in equation (8), leads to the following implicit system of linear equations:

15 $${[\begin{array}{cccc}{I}_{3\times 3} & {A}_{21} & \cdots  & {A}_{N1}\\ {A}_{12} & {I}_{3\times 3} & \cdots  & {A}_{N2}\\ \vdots  & \vdots  & \vdots  & \vdots \\ {A}_{1N} & {A}_{2N} & \cdots  & {I}_{3\times 3}\end{array}]}_{3N\times 3N}{[\begin{array}{c}{v}_{1H}\\ {v}_{2H}\\ \vdots \\ {v}_{NH}\end{array}]}_{3N\times 1}={[\begin{array}{c}{v}_{1}\\ {v}_{2}\\ \vdots \\ {v}_{N}\end{array}]}_{3N\times 1}.$$

This system of equations can then be solved using standard linear algebra methods. The inputs of the system are absolute velocities, *v*_*i*_, which are assigned to individual beads that assemble a rigid body, and outputs are hydrodynamic velocities.

We now validate our numerical method of modeling hydrodynamic interactions using a 3D beads model realization of two nearby rotating disks, where each disk is composed of a large number of beads (see Fig. 6b). The optimum number of beads required to model each disk is determined through the convergence of results. For the presented case in Fig. 6b, as an example, the optimum number of beads is 331, which corresponds to *R*_0_/*a* = 1/21 ≈ 0.05. It should be noted that the thickness (2*R*_0_) of each disk can be neglected compared to its diameter (2*a*), as expected by the swimmer model. Then, the general set of equations (14) must be solved for the entire system of the beads. Drag element exerted on each bead is then determined by multiplying the translational drag coefficient, 6*πμR*_0_, to the bead’s consequent hydrodynamic velocity. At the end, the final results of this three-dimensional simulation is compared to our numerical results of the point force and torque models.

**Figure 7:**
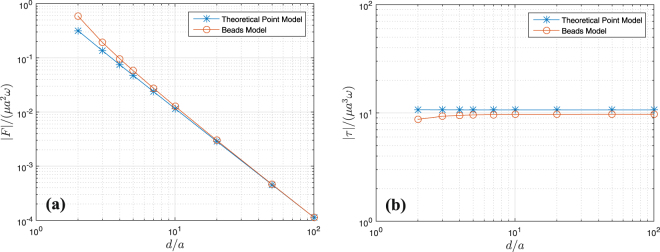
Magnitudes of the total force (**a**) and torque (**b**) exerted on two interacting rotating disks of radius *a* as a function of their distance. The schematics of our setup has been shown in Fig. 6b. We have set *ω* = |*ω*_1_| = |*ω*_2_| = 0.5*ω*_*s*_. Stars and circles respectively correspond to the results of our theoretical (point force and torque) and 3D beads models.

Figure 6b demonstrates the schematics of the problem setup, where two disks of radius *a* and located at a distance of *d* are rotating with angular velocities *ω*_1_ and *ω*_2_. The interaction of these two disks is modeled using: (i) our point force and torque models, and (ii) the full three-dimensional beads simulation. Our results displayed in Fig. 7 show a good agreement between the two models, even for the smallest possible distance, *d* = 2*a*, between the disks. Figure 7 represents the magnitudes of the total force and torque exerted on disks as a function of their distance. The disks are counter-rotating with |*ω*_1_| = |*ω*_2_| = 0.5*ω*_*s*_, and the total exerted force and torque (on each) are computed instantaneously for different distances between them.

## References

[1] Christner BC, Morris CE, Foreman CM, Cai R, Sands DC (2008). Ubiquity of biological ice nucleators in snowfall. Science.

[2] Mooshammer, M. *et al*. Adjustment of microbial nitrogen use efficiency to carbon: nitrogen imbalances regulates soil nitrogen cycling. *Nature Communications* **5** (2014). 10.1038/ncomms4694 PMC3997803 24739236

[3] Locey, K. J. & Lennon, J. T. Scaling laws predict global microbial diversity. *Proceedings of the National Academy of Sciences* 201521291 (2016). 10.1073/pnas.1521291113 PMC4889364 27140646

[4] Elgeti J, Winkler RG, Gompper G (2015). Physics of microswimmers–single particle motion and collective behavior: a review. Reports on Progress in Physics.

[5] Simmchen, J. *et al*. Topographical pathways guide chemical microswimmers. *Nature Communications* **7** (2016). 10.1038/ncomms10598 PMC4748132 26856370

[6] Jeanneret R, Pushkin DO, Kantsler V, Polin M (2016). Entrainment dominates the interaction of microalgae with micron-sized objects. Nature Communications.

[7] Qiu, T. *et al*. Swimming by reciprocal motion at low reynolds number. *Nature Communications* **5** (2014). 10.1038/ncomms6119 PMC4241991 25369018

[8] Wensink HH (2012). Meso-scale turbulence in living fluids. Proceedings of the National Academy of Sciences.

[9] Dunkel J (2013). Fluid dynamics of bacterial turbulence. Physical Review Letters.

[10] Bricard, A. *et al*. Emergent vortices in populations of colloidal rollers. *Nature Communications* **6** (2015). 10.1038/ncomms8470 PMC4557359 26088835

[11] Marconi UMB, Maggi C (2015). Towards a statistical mechanical theory of active fluids. Soft matter.

[12] Alexander GP, Yeomans J (2008). Dumb-bell swimmers. Europhysics Letters.

[13] Lauga E, Bartolo D (2008). No many-scallop theorem: Collective locomotion of reciprocal swimmers. Physical Review E.

[14] Ishikawa T, Sekiya G, Imai Y, Yamaguchi T (2007). Hydrodynamic interactions between two swimming bacteria. Biophysical Journal.

[15] Backholm M, Schulman RD, Ryu WS, Dalnoki-Veress K (2014). Tangling of tethered swimmers: Interactions between two nematodes. Physical Review Letters.

[16] Ishikawa T, Hota M (2006). Interaction of two swimming paramecia. Journal of Experimental Biology.

[17] Ariel, G. *et al*. Swarming bacteria migrate by lévy walk. *Nature Communications* **6** (2015). 10.1038/ncomms9396 PMC4598630 26403719

[18] Ishikawa, T. Suspension biomechanics of swimming microbes. *Journal of The Royal Society Interface* 20090223 (2009). 10.1098/rsif.2009.0223 PMC2838254 19674997

[19] Ishikawa T, Simmonds MP, Pedley TJ (2006). Hydrodynamic interaction of two swimming model micro-organisms. Journal of Fluid Mechanics.

[20] Li G, Ostace A, Ardekani AM (2016). Hydrodynamic interaction of swimming organisms in an inertial regime. Physical Review E.

[21] Michelin S, Lauga E (2010). The long-time dynamics of two hydrodynamically-coupled swimming cells. Bulletin of mathematical biology.

[22] Kim M, Powers TR (2004). Hydrodynamic interactions between rotating helices. Physical review E.

[23] Pooley C, Alexander G, Yeomans J (2007). Hydrodynamic interaction between two swimmers at low reynolds number. Physical Review Letters.

[24] Farzin M, Ronasi K, Najafi A (2012). General aspects of hydrodynamic interactions between three-sphere low-reynolds-number swimmers. Physical Review E.

[25] Alexander GP, Pooley C, Yeomans JM (2008). Scattering of low-reynolds-number swimmers. Physical Review E.

[26] Gilbert AD, Ogrin FY, Petrov PG, Winlove CP (2011). Motion and mixing for multiple ferromagnetic microswimmers. The European Physical Journal E.

[27] Najafi A, Golestanian R (2004). Simple swimmer at low reynolds number: Three linked spheres. Physical Review E.

[28] Klindt GS, Friedrich BM (2015). Flagellar swimmers oscillate between pusher-and puller-type swimming. Physical Review E.

[29] Guasto JS, Johnson KA, Gollub JP (2010). Oscillatory flows induced by microorganisms swimming in two dimensions. Physical Review Letters.

[30] Jalali MA, Khoshnood A, Alam M-R (2015). Microswimmer-induced chaotic mixing. Journal of Fluid Mechanics.

[31] Ratcliff, W. C. *et al*. Experimental evolution of an alternating uni-and multicellular life cycle in chlamydomonas reinhardtii. *Nature Communications* **4** (2013). 10.1038/ncomms3742 PMC3831279 24193369

[32] Koumura N (1999). Light-driven monodirectional molecular rotor. Nature.

[33] Jalali MA, Alam M-R, Mousavi S (2014). Versatile low-reynolds-number swimmer with three-dimensional maneuverability. Physical Review E.

[34] Medina-Sánchez, M., & Schmidt, O. G. Medical microbots need better imaging and control. *Nature* **545**, 406–408 (25 May 2017). 10.1038/545406a 28541344

[35] Nelson BJ, Kaliakatsos IK, Abbott JJ (2010). Microrobots for minimally invasive medicine. Annual review of biomedical engineering.

[36] Or Y, Murray RM (2009). Dynamics and stability of a class of low Reynolds number swimmers near a wall. Physical Review E.

[37] Zhang, S., Or, Y. & Murray, R. M. Experimental demonstration of the dynamics and stability of a low Reynolds number swimmer near a plane wall. *American Control Conference* 4205–4210 (2010).

[38] Mavroidis, C. & Ferreira, A. Nanorobotics: past, present, and future. *Nanorobotics*, 3–27 (2013).

[39] Friedrich BM, Jülicher F (2012). Flagellar synchronization independent of hydrodynamic interactions. Physical Review Letters.

[40] Lopez D, Lauga E (2014). Dynamics of swimming bacteria at complex interfaces. Physics of Fluids.

[41] Kage A, Mogami Y (2015). Individual Flagellar Waveform Affects Collective Behavior of Chlamydomonas reinhardtii. Zoological science.

[42] Drescher K (2009). Dancing volvox: hydrodynamic bound states of swimming algae. Physical Review Letters.

[43] Ben-Jacob E, Cohen I, Levine H (2000). Cooperative self-organization of microorganisms. Advances in Physics.

[44] Peruani F (2012). Collective motion and non-equilibrium cluster formation in colonies of gliding bacteria. Physical Review Letters.

[45] Chandran PL, Mofrad MR (2010). Averaged implicit hydrodynamic model of semiflexible filaments. Physical Review E.

[46] Ota S (2014). Brownian motion of tethered nanowires. Physical Review E.

